# Environmental exposure and HPV infection may act synergistically to induce lung tumorigenesis in nonsmokers

**DOI:** 10.18632/oncotarget.7628

**Published:** 2016-02-23

**Authors:** Ya-Wen Cheng, Frank Cheau-Feng Lin, Chih-Yi Chen, Nan-Yung Hsu

**Affiliations:** ^1^ Graduate Institute of Cancer Biology and Drug Discovery, College of Medical Science and Technology, Taipei Medical University, Taipei, Taiwan; ^2^ Cancer Center, Taipei Medical University Hospital, Taipei Medical University, Taipei, Taiwan; ^3^ School of Medicine, Chung Shan Medical University, Taichung, Taiwan; ^4^ Department of Surgery, Chung Shan Medical University Hospital, Taichung, Taiwan; ^5^ Department of Surgery, Taipei Medical University Hospital, Taipei, Taiwan

**Keywords:** B[a]P, HPV, DNA repair, promoter hypermethylation, environmental exposure

## Abstract

Most studies of lung tumorigenesis have focused on smokers rather than nonsmokers. In this study, we used human papillomavirus (HPV)-positive and HPV-negative lung cancer cells to test the hypothesis that HPV infection synergistically increases DNA damage induced by exposure to the carcinogen benzo[a]pyrene (B[a]P), and contributes to lung tumorigenesis in nonsmokers. DNA adduct levels induced by B[a]P in HPV-positive cells were significantly higher than in HPV-negative cells. The DNA adduct formation was dependent on HPV E6 oncoprotein expression. Gene and protein expression of two DNA repair genes, XRCC3 and XRCC5, were lower in B[a]P-treated E6-positive cells than in E6-negative lung cancer cells. The reduced expression was also detected immunohistochemically and was caused by increased promoter hypermethylation. Moreover, mutations of p53 and epidermal growth factor receptor (EGFR) genes in lung cancer patients were associated with XRCC5 inactivation. In sum, our study indicates that HPV E6-induced promoter hypermethylation of the XRCC3 and XRCC5 DNA repair genes and the resultant decrease in their expression increases B[a]P-induced DNA adducts and contributes to lung tumorigenesis in nonsmokers.

## INTRODUCTION

Lung cancer is a leading cause of cancer-related deaths in Taiwan as well as in other parts of the world [1–2]. The most important risk factor of lung cancer is cigarette smoking [3]. However, there is evidence of increasing incidence of female lung cancer in Taiwan without a concurrent increase in smoking [2, 4]. Thus, it is conceivable that environmental factors other than cigarette smoking may be associated with the development of lung cancer in Taiwan.

Human papillomavirus (HPV) is a well-known cause of cervical cancer, oral cancer, and lung cancer [5]. In a previous study, we observed a high frequency of oncogenic HPV types 16/18 in lung tumor tissues of nonsmoking females [6]. In addition, the HPV16/18 E6 protein, which inactivates the tumor suppressor protein p53, was detected in about half of HPV16/18-positive lung tumors [7]. A previous report indicated that inactivation of p53 might induce chromosome instability associated with tumor progression [8]. We also observed a higher-frequency loss of heterozygosity (LOH) of the fragile histidine triad gene in HPV 16-infected lung tumors of females, suggesting that the HPV involvement in lung tumorigenesis may be mediated through induced chromosome instability [9].

Polycyclic aromatic hydrocarbons, such as benzo(a) pyrene (B[a]P), are known causes of lung cancer [10,11]. They exist in cigarettes, cooking oil, roast smoke, and air pollution [12, 13]. B[a]P may be metabolized to benzo[a]pyrene 7,8-diol 9,10-epoxide (BPDE) and adducted to DNA to induce a G-to-T transversion mutation of the p53 gene [14] or chromosome instability [15]. We have previously detected higher levels of BPDE-DNA adducts in female nonsmokers compared with smoking and nonsmoking male lung cancer patients [16].

Studies have reported promoter hypermethylation in virus-induced carcinogenesis, including hepatoma and cervical cancer [17,18]. Increased promoter hypermethylation was also found in HPV-induced lung cancer [19,20]. We have previously observed that DNA (cytosine-5)-methyltransferase 3 beta (DNMT3b) protein expression was induced in HPV-infected lung tumors, resulting in increased promoter hypermethylation of the p16 gene [20]. Gene promoter hypermethylation was also increased after treatment with B[a]P, and was mediated by DNA (cytosine-5)-methyltransferase 1 (DNMT1), resulting in the transformation of immortalized bronchial epithelial cells [21]. A previous report indicated that promoter hypermethylation is the main pathway involved in repair gene inactivation [22]. In another study, inactivation of repair genes, combined with exposure to B[a]P, had a synergistic effect on DNA damage [23].

Previous reports have showed that HPV infection inhibits expression of repair genes including hMLH1, hMSH2, XRCC1, XRCC3, XRCC5, BRCA1, BRCA2, MGMT, ERCC1, ERCC2, and ERCC4 [24–29]. Our previous study has also indicated that the HPV16/18 E6 oncoprotein may contribute to EGFR mutations through inhibited hMLH1 and hMSH2 gene expression in Taiwanese lung cancer patients [30]. In addition, Tung et al., (2015) demonstrated that B[a]P exposure reduced the expression of XRCC5, p53, and DNA-PK in mouse lung tissue [31].

In this study, we hypothesized that HPV and B[a]P work together to damage the lung DNA by inactivating DNA repair genes and inducing their promoter hypermethylation. We used HPV-infected and non-infected lung cancer cell lines and lung tumor tissues to examine the association of the HPV16/18 E6 protein, p53, promoter hypermethylation of repair genes, expression of repair genes, and BPDE-like DNA adduct levels.

## RESULTS

### HPV-infected lung cancer cells exhibit higher BPDE-like DNA adduct levels than HPV-negative cells after low-dose B[a]P treatment

**Figure 1:**
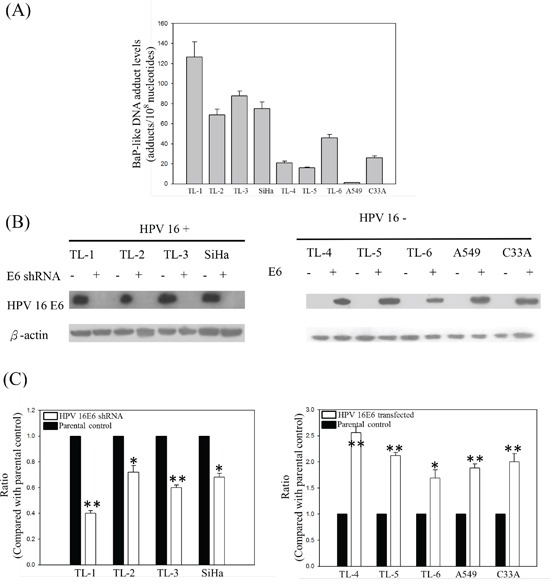
**A.** BPDE-like DNA adduct levels in HPV-positive and -negative lung cancer cell lines treated with low-dose B[a]P. **B.** and **C.** BPDE-like DNA adduct levels in HPV 16 E6 knockdown or transfection HPV-positive (TL-1 to TL-3 and SiHa) and HPV-negative (TL-4 to TL-6, A549 and C33A) cancer cell lines. The cells were treated with 0.1μM B[a]P for 7 days. Data are the results from three individual experiments (* p<0.05; ** p<0.001, compared with parental control).

We have previously detected high HPV infection rates and high BPDE-like DNA adduct levels in female nonsmoking lung cancer patients [13]. To determine whether the HPV-infected lung tumor cells were more sensitive to B[a]P exposure than the noninfected ones, HPV16-positive and -negative lung cancer cells were treated with low-dose B[a]P, and the DNA adduct levels were detected by ELISA. After treatment with 0.1 μM B[a]P for 7 days, the BPDE-like DNA adduct levels in the HPV16-positive lung cancer cell lines (TL-1 to TL-3 and SiHa) were significantly higher than in the HPV 16-negative cell lines (TL-4 to TL-6, A549 and C33A; Figure 1A). After knockdown of HPV16 E6 (Figure 1B), the BPDE-like DNA adduct levels decreased compared with those of the parental cells (*p* < 0.05; Figure 1C). The reverse correlation was found in HPV16 E6-transfected HPV-negative lung cancer cells and their parental controls (*p* < 0.001; Figure 1C). Thus, we considered that HPV infection may increase BPDE-like DNA adduct formation or decrease DNA repair capability after exposure to B[a]P.

### Treatment with low-dose B[a]P suppresses expression of repair genes XRCC3 and XRCC5 in HPV-infected lung cancer cells

**Figure 2:**
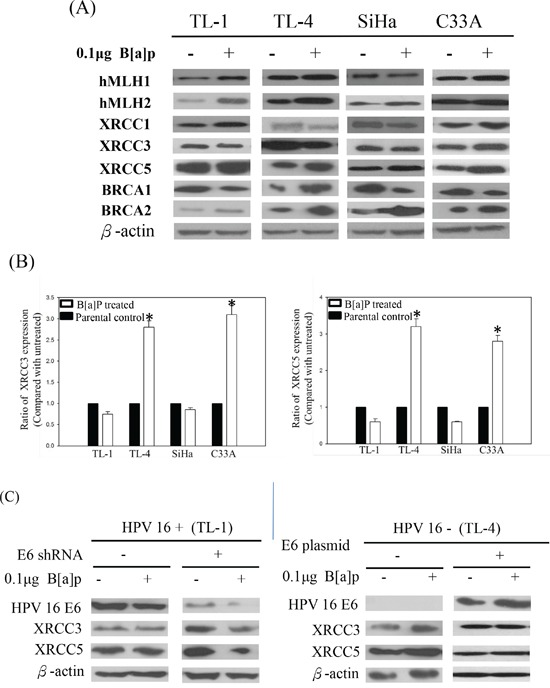
**A.** Representative protein expression of repair genes in HPV-positive (TL-1 and SiHa) and HPV-negative (TL-4 and C33A) cancer cells after B[a]P treatment. **B.** Changes of XRCC3 and XRCC5 mRNA expression in HPV-positive and HPV-negative cancer cells after B[a]P treatment compared with parental control (*p < 0.001, compared with parental control). **C.** Protein levels of XRCC3 and XRCC5 in response to B[a]P treatment after knockdown or transfection of HPV16 E6 in HPV-positive and HPV-negative lung cancer cells.

Suppression of the repair genes correlates with the formation of BPDE-like DNA adducts [23]. To understand the role of repair genes in the formation of BPDE-like DNA adducts in HPV-associated lung tumor progression, we analyzed their expression in the B[a]P-treated HPV-positive and -negative lung cancer cells. The protein expression of hMLH1, hMSH2, XRCC1, XRCC3, XRCC5, BRCA1, and BRCA2 repair genes in B[a]P-treated HPV-positive and -negative lung cancer cells was analyzed by western blotting. As shown in Figure 2A, only XRCC3 and XRCC5 were not induced in HPV-positive TL-1 and SiHa cells after treatment with B[a]P. XRCC3 and XRCC5 mRNA levels were decreased in the HPV-positive TL-1 and SiHa cells after B[a]P treatment (Figure 2B). In contrast to HPV-positive cells, B[a]P treatment increased XRCC3 and XRCC5 protein and mRNA levels in the HPV-negative TL-4 and C33A cells (Figures 2A and 2B). After knockdown of HPV16 E6 in HPV-positive lung cancer TL-1 cells, the expression of XRCC3 and XRCC5 genes was increased in response to the B[a]P treatment compared with their parental cells (Figure 2C). These reversed associations were also found in HPV 16 E6-transfected HPV-negative lung cancer TL-4 cells (Figure 2C, right panel). Thus, we concluded that HPV infection might inhibit XRCC3 and XRCC5 gene expression after exposure to B[a]P, decreasing the repair ability of these genes.

### Promoter hypermethylation suppresses the expression of XRCC3 and XRCC5 via DNMT1 and DNMT3b overexpression

**Figure 3:**
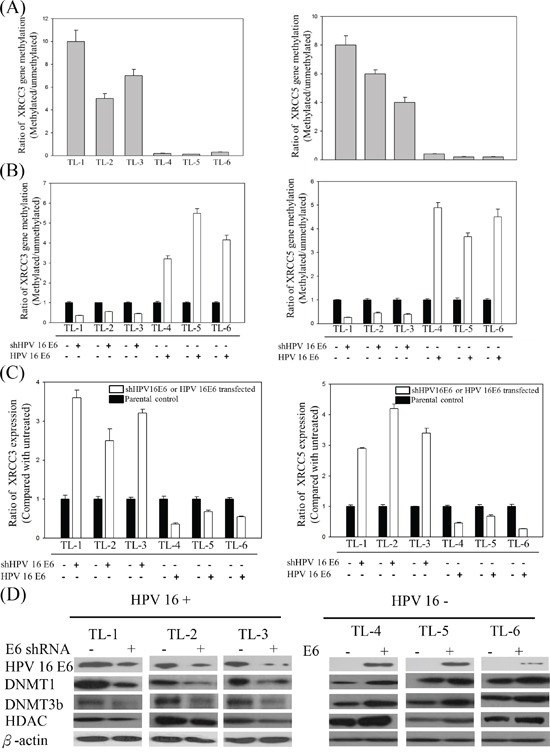
**A.** Methylation levels of XRCC3 and XRCC5 in B[a]P-treated HPV16-positive and -negative lung cancer lines. **B.** XRCC3 and XRCC5 promoter hypermethylation and **C.** mRNA expression in response to the B[a]P treatment after knockdown or transfection of HPV16 E6 in HPV-positive and HPV-negative lung cancer cells. **D.** Representative protein levels of DNA methylation-associated genes, including DNMT1, DNMT3b, and HDAC, analyzed by Western blotting in HPV-positive and -negative lung cancer cell lines after HPV16 E6 knockdown or transfection.

In a previous study, we demonstrated that the expression of DNMT3b in HPV-positive lung tumors was higher than that in HPV-negative tumors [19]. To determine whether promoter hypermethylation caused the inhibition of XRCC3 and XRCC5 expression in the HPV16-positive lung cancer cells, we analyzed the methylation status of XRCC3 and XRCC5 and the expression of the methylation-associated proteins histone deacetylases (HDAC), DNMT1, and DNMT3b in B[a]P-treated HPV-positive and -negative lung cancer cells. As shown in Figure 3, the methylation levels of XRCC3 and XRCC5 in the HPV16-positive TL-1, TL-2, and TL-3 lines were significantly higher than in the HPV16 E6-negative TL-4, TL-5, and TL-6 cells. In the HPV-positive lung cancer cells, after knockdown HPV16 E6 expression, the promoter hypermethylation of the XRCC3 and XRCC5 genes was decreased. The reversed results were observed in HPV-negative cell lines (Figure 3B). In addition, the mRNA expression levels of XRCC3 and XRCC5 were inversely correlated with promoter methylation (Figure 3C). These results suggest that HPV16 E6 enhances hypermethylation. To determine which methylation-associated proteins were involved in HPV-induced methylation, the expression of the DNMT1, DNMT3b, and HDAC proteins was analyzed by Western blot. As shown in Figure 3D, protein levels of DNMT1, DNMT3b, and HDAC in the HPV16-positive or HPV16 E6-transfected lung cancer cells were higher than in the HPV-negative or HPV16 E6 knockdown cells.

### HPV E6 induces DNMT1 and DNMT3b gene expression via inhibition of p53 protein expression

**Figure 4:**
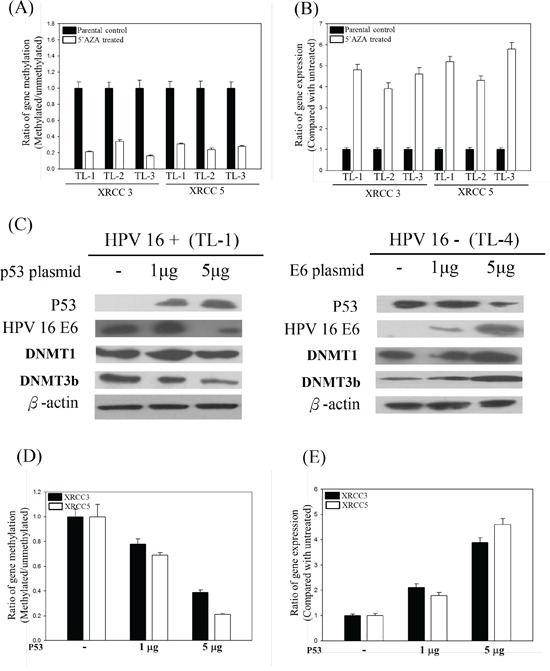
**A.** XRCC3 and XRCC5 promoter methylation and **B.** XRCC3 and XRCC5 gene expression levels in 5′-AZA-treated HPV-positive and -negative lung cancer cells. **C.** Representative protein levels of DNA methylation-associated genes, including DNMT1, DNMT3b, and HDAC, analyzed by Western blotting in HPV-positive or -negative lung cancer cell lines after p53 or HPV 16 E6 transfection. **D.** Promoter methylation and **E.** mRNA levels of XRCC3 and XRCC5 in p53-transfected HPV-positive (TL-1) lung cancer cells.

We demonstrated the inactivation of the p53 protein in lung tumor tissues by HPV16 E6 in a previous report [7]. In addition, we found that p53 could regulate promoter methylation in lung cancer [19, 20]. 5′-AZA is known to inhibit three DNMTs and to stabilize p53 [32]. After treatment with 5′-AZA, XRCC3 and XRCC5 promoter methylation was clearly reduced and, consequently, levels of mRNA were partially recovered in these cells (Figures 4A and 4B). Therefore, we investigated whether transfection with different amounts of WT-p53 into HPV16-positive lung cancer cells would modulate the promoter methylation of XRCC3 and XRCC5 genes and their mRNA levels. The p53 protein levels in the transfected cells were increased with increasing amounts of the WT-p53 construct (Figure 4C). The promoter methylation status of XRCC3 and XRCC5 gradually decreased with increasing amounts of the transfected WT-p53 (Figure 4D), and the XRCC3 and XRCC5 mRNA levels consequently increased with increasing levels of WT-p53 protein (Figure 4E). In addition, after transfection, HPV16 E6 inhibited the function of the p53 protein, and the promoter hypermethylation of XRCC3 and XRCC5 increased (Figures 4D and 4E). These results indicate that HPV16 E6 induces DNMT1 and DNMT3b gene expression via inhibition of p53 expression.

### HPV16 E6-positive lung tumor tissues and low XRCC3 protein expression are associated with high BPDE-DNA adduct levels

We analyzed the association of HPV infection, XRCC3/XRCC5 expression, and BPDE-DNA adduct levels in lung tumor tissues. As shown in Table 1, the samples were grouped as XRCC3-/HPV16 E6+, XRCC3-/HPV16 E6-, XRCC3+/HPV16 E6+, and XRCC3+/HPV16 E6- to compare the DNA adduct levels. High DNA adduct levels were detected in the XRCC3-/HPV16 E6+ group compared to the other three groups (*p* = 0.007; Table 1). The XRCC5-/HPV16 E6+ group also showed higher DNA adduct levels compared to the other three groups (*p* < 0.0001).

**Table 1:** XRCC3 and XRCC5 protein expression in tumor tissues from lung cancer patients with different HPV-infection and BPDE-like DNA-adduct levels

DNA adduct levels/HPV infection	Protein expression		*p*-value
	Negative (%)	Positive (%)	
**XRCC3**			
High/+ (n = 24)	15 (62.5)	9 (37.5)	
High/− (n = 44)	11 (25.0)	33 (75.0)	
Low/+ (n = 6)	3(50.0)	3 (50.0)	
Low/− (n = 26)	6 (23.1)	20 (76.9)	0.007
**XRCC5**			
High/+ (n = 24)	14 (58.3)	10 (41.7)	
High/− (n = 44)	6 (13.6)	38 (86.4)	
Low/+ (n = 6)	6 (100.0)	0 (0.0)	
Low/− (n = 26)	9 (34.6)	17 (65.4)	<0.0001

### Inactivation of the XRCC5 protein is associated with EGFR and p53 gene mutations in lung tumor tissues

Results from the lung tumor tissues revealed that suppression of XRCC5 was associated with p53 and EGFR mutations. The p53 and EGFR mutations were more frequent in patients with low XRCC5 protein levels than in those with high XRCC5 expression (p = 0.016 for p53; p = 0.003 for EGFR). However, there was no correlation between XRCC3 protein expression, and EGFR and p53 gene mutations (Table 2). In addition, the frequency of p53 and EGFR gene mutations was higher in the XRCC5 group with a high level of DNA adducts than in the other three groups. The association was also found in patients grouped by XRCC3 and DNA adduct levels (Table 2). The results suggest that after exposure to B[a]P, promoter hypermethylation represses the expression of XRCC5 in HPV16-infected lung tissue. The suppression of repair genes may encourage the accumulation of DNA adducts and contribute to lung tumorigenesis in nonsmokers.

**Table 2:** XRCC3, XRCC5, BPDE-like DNA adduct levels, and EGFR/p53 mutation in lung tumor tissues of lung cancer patients

Gene	p53			EGFR		
	Wild type		Mutant type	Wild type		Mutant type
XRCC3						
Low	19		16	15		20
High	46		19	40		25
*P* value		0.125			0.093	
XRCC5						
Low	17		18	12		23
High	48		17	43		22
*P* value		0.016			0.003	
XRCC3/DNA adduct levels						
Low/low	9		0	7		2
Low/high	10		16	8		18
High/low	15		8	19		4
High/high	31		11	21		21
*P* value		0.001			0.001	
XRCC5/DNA adduct levels						
Low/low	12		3	9	6
Low/high	5		15	3		17
High/low	12		5	17		0
High/high	36		12	26		22
*P* value		0.001			<0.0001	

### HPV 16 E6 oncoprotein expression correlates with EGFR and p53 gene mutations in lung cancer patients

A number of variables, including XRCC3, XRCC5, DNA adduct levels, and HPV 16 E6 were examined for their effects on EGFR and p53 gene mutations in lung tissues by multivariate logistic regression analysis. Patients with XRCC3/XRCC5 low protein expression had approximately 4.953 and 19.2-fold increased risk of EGFR gene mutation when compared with patients with high XRCC3/XRCC5 protein expression (95% confidence interval (CI) = 1.045 - 23.482 for XRCC3, P = 0.044; 95% CI = 3.012 - 125, P = 0.002 for XRCC5). The patients who had high DNA adduct levels had an approximately 20.901-fold increased risk of EFGR gene mutation when compared with patients who had low DNA adduct levels (95% CI = 4.065 - 107.480, P < 0.0001). The patients who were HPV 16 E6 positive had an approximately 5.534-fold increased risk of EFGR gene mutation when compared with patients who were HPV 16/18 E6 negative (95% CI = 1.619 - 18.917, P = 0.006). The patients who were HPV 16 E6 positive had an approximately 21.707-fold increased risk of p53 gene mutation when compared with patients who were HPV 16/18 E6 negative (95% CI = 5.859 - 80.420, P < 0.0001). The odds ratios of the other variables did not reach statistical significance (Table 3). Thus, XRCC3, XRCC5, DNA adduct levels, and HPV 16 E6 oncoprotein levels were contributors to EGFR gene mutation in lung cancer patients. In addition, HPV 16 E6 oncoprotein expression correlated with p53 gene mutation in lung cancer patients.

**Table 3:** Multi-variant logistic regression analysis of the risk of EGFR and *p53* genes mutation in lung cancer patients

Parameters	Favorable/ unfavorable	OR	95% CI	p value
***EGFR gene***				
XRCC3	Positive / negative	4.953	1.045 - 23.482	0.044
XRCC5	Positive / negative	19.2	3.012 - 125	0.002
DNA adduct levels	Low/high	20.901	4.065 - 107.480	<0.0001
HPV 16/18 E6 protein	Negative/positive	5.534	1.619 - 18.917	0.006
***P53 gene***				
XRCC3	Positive / negative	2.001	0.379 - 10.564	0.414
XRCC5	Positive / negative	1.567	0.271 - 9.091	0.615
DNA adduct levels	Low/high	1.576	0.437 - 5.680	0.487
HPV 16/18 E6 protein	Negative/positive	21.707	5.859 - 80.42	<0.0001

## DISCUSSION

Epidemiological and clinical data have provided support for an association of HPV infection with several types of cancer [5, 6, 33–37]. However, HPV infection alone appears to be insufficient for cancer development. A previous study showed that the majority of HPV infection is cleared by the immune response within 8–12 months [37]. Another study demonstrated that additional cofactors are needed for prolonged expression of the HPV oncoprotein in the development and progression of cervical cancer [38]. In addition, cigarette smoke was reported to have a late-stage synergistic effect on cervical carcinogenesis initiated by HPV infection [39]. In previous studies, we observed a high prevalence of HPV16/18 infection and high DNA adduct levels in the tumor tissues of female lung cancer patients [6,16]. In the present study, we found that BPDE-like DNA adduct levels in HPV-infected lung cancer patients were significantly higher than in non-infected ones. Thus, we suggest that B[a]P exposure and HPV infection may exert a synergistic effect on lung tumorigenesis in nonsmoking females in Taiwan.

A previous report found that human bronchial epithelial (HBE) cells with a defect in ERCC1, ERCC2, ATM, or MSH2 DNA repair pathway genes were more sensitive to B[a]P-induced DNA damage [40]. The same study failed to observe enhanced effects of B[a]P exposure on the induction of cell transformation after treatment of up to 20 weeks. These data indicate that a deficiency of DNA repair pathway genes alone does not shorten the latency of cell transformation. The authors also found that HBE cells expressing H-Ras or c-Myc were transformed 8–12 weeks after B[a]P treatment [40]. Thus, they proposed that the silencing of a single DNA repair gene does not make cells susceptible to chemical-induced cell transformation. Another report indicated that exposure to a low concentration (0.1 μm) of B[a]P correlated with HPV genome amplification, which could potentially result in increased templates from which E6 and E7 oncogene transcripts are produced [34]. Increased oncogene expression was also directly correlated with increased carcinogenic potential of the tissue in another study [41]. We showed in a previous report that the expression of HPV16 E6 is associated with lung tumorigenesis [7]. In the present study, we also found that HPV16 E6 protein expression in low-dose B[a]P-exposed lung cancer cells was higher than that in non-exposed cells. In addition, our previous reports found that cyclin D1, c-Myc and hTERT protein expression levels in HPV16/18 E6-positive lung tumors were significantly higher than in HPV16/18 E6-negative tumors [42,43], suggesting that HPV16/18 infection can activate the expression of oncogenes, such as c-myc, cyclinD1and hTERT. Thus, we suggest that low-dose B[a]P exposure may induce HPV16 E6 protein expression and c-myc oncoprotein expression, resulting in lung cell transformation.

In a previous report, we showed that the HPV16 E6 oncoprotein could decrease p16 and tissue inhibitor of MMP-3 (metalloproteinases-3) gene expression through promoter hypermethylation by inducing DNMT 1 and DNMT3b protein expression [19,20,44]. In this study, we also found that HPV infection could repress XRCC3 and XRCC5 repair gene expression via induction of promoter hypermethylation, resulting in high DNA adduct levels in lung tumor tissues. High DNA adduct accumulation was associated with gene mutation. High DNA adduct levels were detected in the XRCC3-/HPV16 E6+ group but not in the other three groups (Table 1). Higher DNA adduct levels were also detected in the XRCC5-/HPV16 E6+ group than in the other three groups (Table 1). In addition, the frequency of the p53 and EGFR mutations was higher in patients with low XRCC5 protein expression than in those with high XRCC5 expression. Therefore, we suggest that HPV16 infection may be associated with decreased repair capacity, resulting in DNA adduct accumulation and the induction of gene mutations in lung pathogenesis.

Our previous study indicated that BPDE-like DNA adduct levels in lung tissues from smoking lung cancer patients were not higher than those of nonsmoking patients [38]. We also observed that the adduct levels in nonsmoking female lung cancer patients were higher than in nonsmoking male patients, suggesting that females may be more susceptible to environmental carcinogen exposure than males [38]. In addition, we observed a high prevalence of HPV16/18 infection in female lung cancer patients compared to male patients [6]. Moreover, our recent report further indicated that HPV16/18 E6 oncoprotein is expressed in lung tumors and related with p53 inactivation, strongly suggesting that HPV16/18 infection may be involved in lung tumor formation, at least in Taiwanese women nonsmokers [7]. In the present study, we have found that BPDE-like DNA adduct levels in HPV-infected lung cancer cells are higher than in HPV-negative cells after B[a]P exposure (Figure 1). Thus, these results suggest that the combined effects of B[a]P-induced DNA damage and HPV infection could synergistically induce lung tumor formation, especially in female patients.

In conclusion, in this study, we have found that HPV and B[a]P work together to confer DNA damage in lungs by increasing promoter hypermethylation and reducing expression of DNA repair genes.

## MATERIALS AND METHODS

### Study subjects

The study was approved by the Institutional Review Board. Primary lung cancer patients who were admitted to the Department of Thoracic Surgery, Chung Shan Medical University, Taichung, Taiwan, between 2000 and 2010 provided written informed consent. None of the subjects had received radiation therapy or chemotherapy prior to surgery. Some of the collected lung tumors had been previously analyzed for the presence of HPV16 and/or 18 DNA [6] and E6 protein [7]. Tumor types and stages were histologically determined according to the World Health Organization classification (World Health Organization, 2012). Pathological material was processed for conventional histological procedures. 100 primary lung cancer patients including 45 female and 55 male. All female lung cancer patients are life-time never smoker. In 55 male patients, including 30 smokers and 25 non-smokers. Information on smoking history was obtained from the patients by interview with informed consent. Smokers and non-smokers were current smokers who smoked up to the day of pulmonary surgery and life-time non-smokers, respectively.

### Cell culture

Cell lines of non-small cell lung cancer with and without HPV infection were established from malignant pleural effusion of lung cancer patients [7]. Lung cancer cell line TL-1, TL-2, and TL-3 are HPV16-infected adenocarcinoma; TL-4, TL-5, and TL-6 are HPV non-infected adenocarcinoma. The cells were confirmed as lung cancer cells by karyotyping and immunoreactivity using thyroid transcription factor-1 antibody. HPV infection was verified by FISH, nested PCR, RT-PCR, and Western blotting analysis of HPV16/18 DNA, E6 mRNA, and E6 protein expression, respectively. A549, SiHa, and C33A cells were purchased from the American Type Culture Collection. Cells were cultured in DMEM, RPMI 1640, or minimum essential medium and supplemented with 10% fetal bovine serum (FBS), 100 U/ml penicillin, and 100 μg/ml streptomycin in a humidified atmosphere with 5% CO_2_ at 37° C.

B[a]P (Sigma-Aldrich, San Diego, CA) was added to culture medium at a final concentration of 0.01 μM, and cells were incubated for 14 days. The medium with freshly added B[a]P was changed every 2 days.

### Silencing of endogenous HPV16 E6 expression by RNA interference

The target sequences for RNA interference (RNAi) for HPV16 E6 were described previously [7]. The sequence of siE6-1 sense strand–directed small interfering RNA (siRNA) was 5′-GAGGUAUAUGACUUUGCUUdTdT-3′ and that of the siE6-2 sense strand–directed siRNA was 5′-GAAUGUGUGUACUGCAAGCdTdT-3′. To suppress transcription of the endogenous HPV16 E6 gene, SiHa and TL-1 cells were transiently transfected with synthetic siRNAs against HPV16 E6 using oligofectamine reagent (Invitrogen, Carlsbad, CA) according to the manufacturer's instructions. Briefly, 24 h before transfection, 1.4 × 10^5^ cells were seeded in each well of a six-well plate. Oligofectamine (3 ml) was added to 12 ml of OPTIMEM (Invitrogen, Carlsbad, CA). After 5 min, 60 pmol of each siRNA in 175 ml of OPTIMEM were combined with the oligofectamine mixture. After incubation for 20 min at 25° C, the siRNA-oligofectamine mixture was added to the cells. After 48 h of incubation at 37°C, the cells were harvested and subjected to real-time quantitative RT-PCR and Western blot analysis.

### Transfection of HPV16 E6 into lung cancer cells

Full-length E6 of HPV16 was amplified by PCR from CasKi cells containing the viral genome of HPV16. The resulting PCR products were purified with a GENECLEAN III kit (MP Biomedicals, Vista, CA). Purified fragments were cloned into a eukaryotic expression vector, pcDNA3.1/V5-His TOPO TA expression kit (Invitrogen, Carlsbad, CA), and the resultant recombinants were transfected into HPV non-infected lung cancer cell lines. On the day prior to transfection, the cells were seeded at 1 × 10^5^ cells per well. After incubation overnight, the cells (30%–50% confluent) were washed twice with phenol red-free DMEM or RPMI 1640 medium without FBS. After incubation with 1 mL of phenol red-free DMEM or RPMI 1640 medium with 10% FBS for 3 h, calcium chloride-Hepes-buffer saline and recombinant DNA solution were added dropwise to the medium in each well of the plate. The cells were then returned to the incubator. After 4 h, the medium was aspirated and the cells were shocked with glycerol solution for 30 sec and then washed twice with PBS. Stable transfectants were selected by culturing transfected cells in the medium containing the antibiotic G418.

### Methylation-specific PCR

Methylation-specific PCR (MSP) was used to determine the hypermethylation status of the promoters of the DNA repair genes as described previously [19, 20]. In brief, DNA was extracted from lung cancer cells treated with B[a]P for 14 days with and without HPV infection. The extracted DNA was purified and then treated with sodium bisulfite (Sigma-Aldrich, San Diego, CA) to convert cytosine to uracil. Methylated cytosine cannot be converted. For each DNA repair gene, we used two sets of primers (one methylated, one not) for PCR amplification. The DNA was then sequenced by ABI 3100 to determine the presence of promoter hypermethylation.

### RNA isolation and real-time RT-PCR

Total RNA from lung tumors (100 mg) and from tumor cell lines (1 × 10^5^ cells) was extracted by homogenization in 1 mL of TRIzol reagent, followed by chloroform re-extraction and isopropanol precipitation. Total RNA (3 μg) was reverse transcribed using SuperScript II Reverse Transcriptase (Invitrogen, Carlsbad, CA) and oligo d(T)15 primers. Real-time quantitative RT-PCR was performed in a final volume of 25 μl containing 1 μl of each cDNA template, 10 pmol of repair gene-specific primers, and 12.5 μl of a SYBR-Green master mix. The primers were designed using ABI Prism 7500 SDS software(**Life Technologies Co., Taiwan)**. Quantification was carried out using the comparative threshold cycle (CT) method. CT values were calculated by determining the cycle number at which the fluorescence exceeded the threshold limit. The average CT values for the target gene were normalized to an endogenous housekeeping gene encoding 18S rRNA.

### Western blot

Protein was extracted from lung cancer colonies with and without HPV infection after treatment with B[a]P for 7 days. The cells were treated with lysis buffer (100 mmol/L Tris pH 8.0 and 1% SDS). Protein concentrations were determined using the Bio-Rad protein assay kit (Bio-Rad Laboratories, Hercules, CA). A total of 20 μg protein was separated by electrophoresis on an SDS-PAGE (12.5% gel, 1.5 mm thick) and then transferred to a polyvinylidene difluoride membrane and blocked in 5% skim milk containing 0.1% TBS-Tween for 20–60 min. Membranes were incubated with primary antibodies for 60 min. The membranes were washed three times with 5% skim milk solution containing 0.1% TBS-Tween 20. The membranes were then incubated 60 min with peroxidase-conjugated secondary antibody (1:5,000 dilution), washed, and protein bands were visualized by enhanced chemiluminescence (NEN Life Science Products, Inc., Boston, MA).

### Immunohistochemistry

Formalin-fixed and paraffin-embedded specimens were sectioned at a thickness of 3 μm. All sections were then deparaffinized in xylene, rehydrated through serial dilutions of alcohol, and washed in PBS (pH 7.2). The same buffer was used for all subsequent washes. For HPV 16 E6, XRCC3, and XRCC5 detection, the sections were heated in a microwave oven twice for 5 min in citrate buffer (pH 6.0) and then incubated with polyclonal anti-HPV 16 E6, anti-XRCC3, and anti-XRCC5 antibodies (Santa Cruz Biotechnology, Santa Cruz, CA) for 90 min at 25° C. The conventional streptavidin peroxidase method (DAKO, LSAB kit K675, Copenhagen, Denmark) was performed for signal amplification, and the cells were counterstained with hematoxylin. Negative controls were obtained by leaving out the primary antibody. Three observers independently evaluated the intensities of the signals. Cells that were 0%–10% positive were defined as negative immunostaining, and those that were more than 10% positive were defined as positive immunostaining. Cervical cancer tumor tissues with HPV16/18 were used as a positive control for HPV16/18 E6.

### Competitive color ELISA analysis of BPDE adduct levels

The BPDE adduct levels were analyzed using rabbit anti-BPDE-DNA antibody as described previously [16]. Briefly, DNA was extracted from lung cancer cells and tumor tissues. BPDE-DNA was coated in the plate. Rabbit anti-BPDE-DNA antibody and extracted DNA were incubated in the plate. The antibody that does not react with the extracted DNA attaches to the plate. After washing and incubation with goat anti-rabbit IgG alkaline phosphatase conjugate, the color was developed by the addition of *p*-nitrophenyl phosphate and measured by a microtiter plate reader (Model 550; Bio-Rad, Hercules, CA).

### Statistical analysis

The χ2 test, Fisher's exact test (two-tailed), and the Mann–Whitney *U* test were used for statistical analysis. All analyses were performed using SPSS version 11.0 (SPSS Inc., Chicago, IL).
